# Coil embolization of saccular frontopolar artery aneurysm

**DOI:** 10.1259/bjrcr.20180016

**Published:** 2018-06-01

**Authors:** Saima Ahmad, Umair Rashid Chaudhry

**Affiliations:** 1 Department of Radiology, Lahore General Hospital, Lahore, Pakistan; 2 Department of Neuroradiology, Lahore General hospital, Lahore, Pakistan

## Abstract

In this case report, a distal anterior cerebral artery aneurysm was presented. While most distal anterior aneurysms occur at the bifurcation of the pericallosal and callosomarginal arteries, this particular aneurysm occurred at the frontopolar artery, a case rarely reported in published literature. The aneurysm had a recurrent subarachnoid haemorrhage due to the rupture of a saccular aneurysm and was treated through endovascular coiling.

## Introduction

We define the distal anterior cerebral artery as the distal portion after the anterior communicating artery. Aneurysms in the distal anterior cerebral artery have low occurrence rates with 2.1–9.2% including both the pericallosal and callosomarginal arteries.^1, 2^ High surgical morbidity is found in the surgical clipping of distal anterior cerebral artery (DACA) aneurysms, when it is done using an interhemispheric approach due to the narrowing of operative space and the necessary sacrifice of bridging veins.^3, 4^

To this end, an endovascular approach is increasingly being used for the treatment of both ruptured and unruptured aneurysms as a primary treatment option when dealing with intracranial aneurysms after the success of the International Subarachnoid Aneurysmal Trial. That said, it is still challenging to treat distal anterior cerebral artery aneurysms using endovascular approach with morbidity results similar to that of clipping.^5^

## Case Report

**Figure 1.**
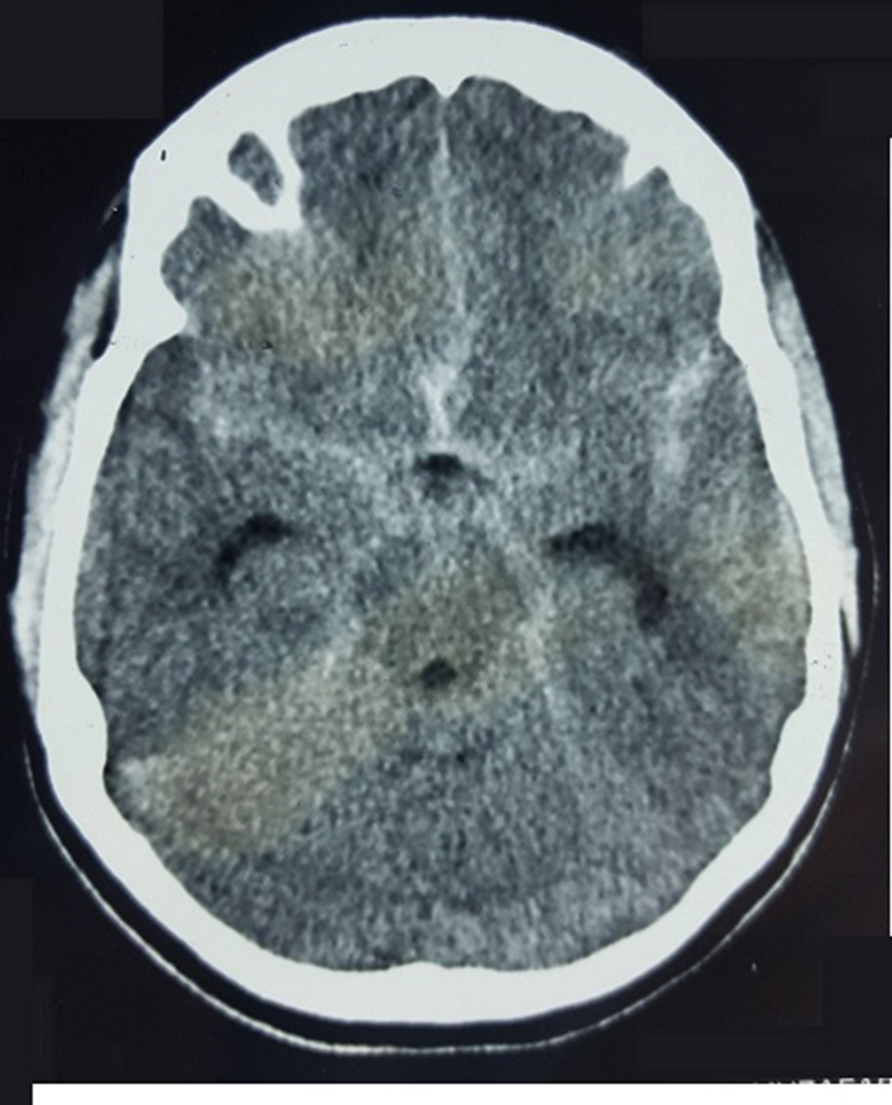
Initial axial CT scan, demonstrating diffuse subarachnoid haemorrhage and cisternal clot at level of interpeduncular cistern.

**Figure 2.**
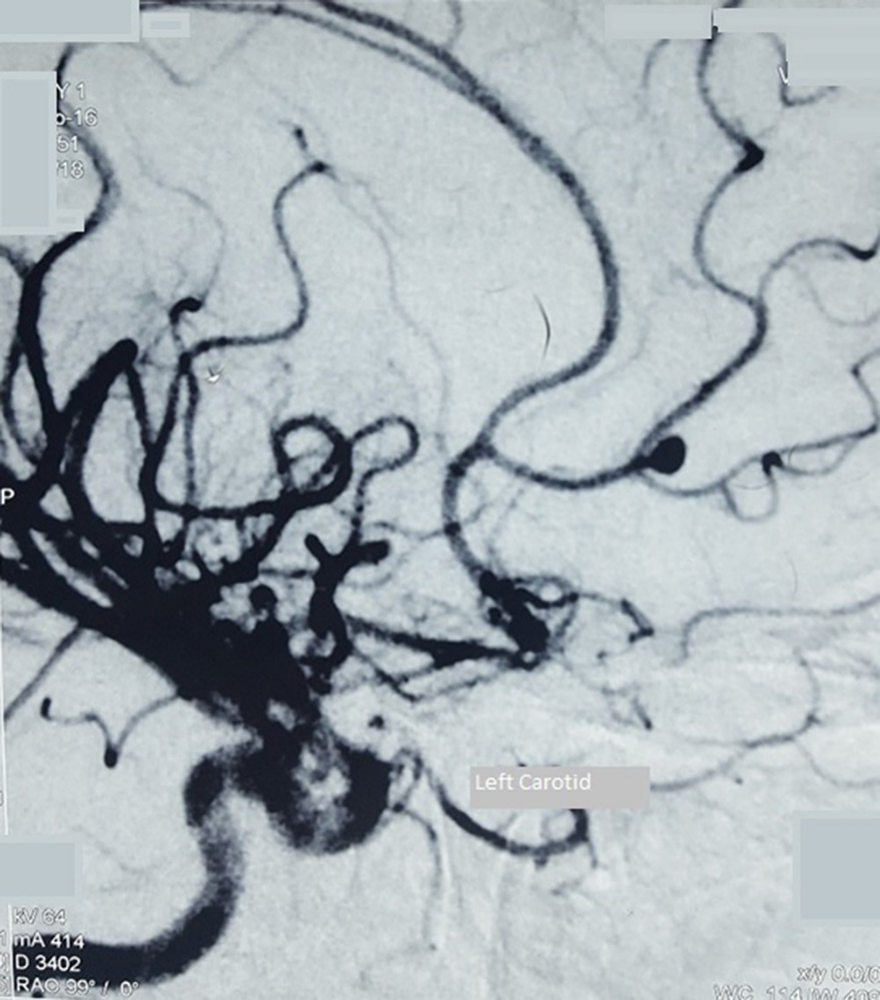
Anteroposterior and lateral view of left carotid angiogram obtained before embolization showing right FPA aneurysm. FPA, frontopolar artery.

**Figure 3.**
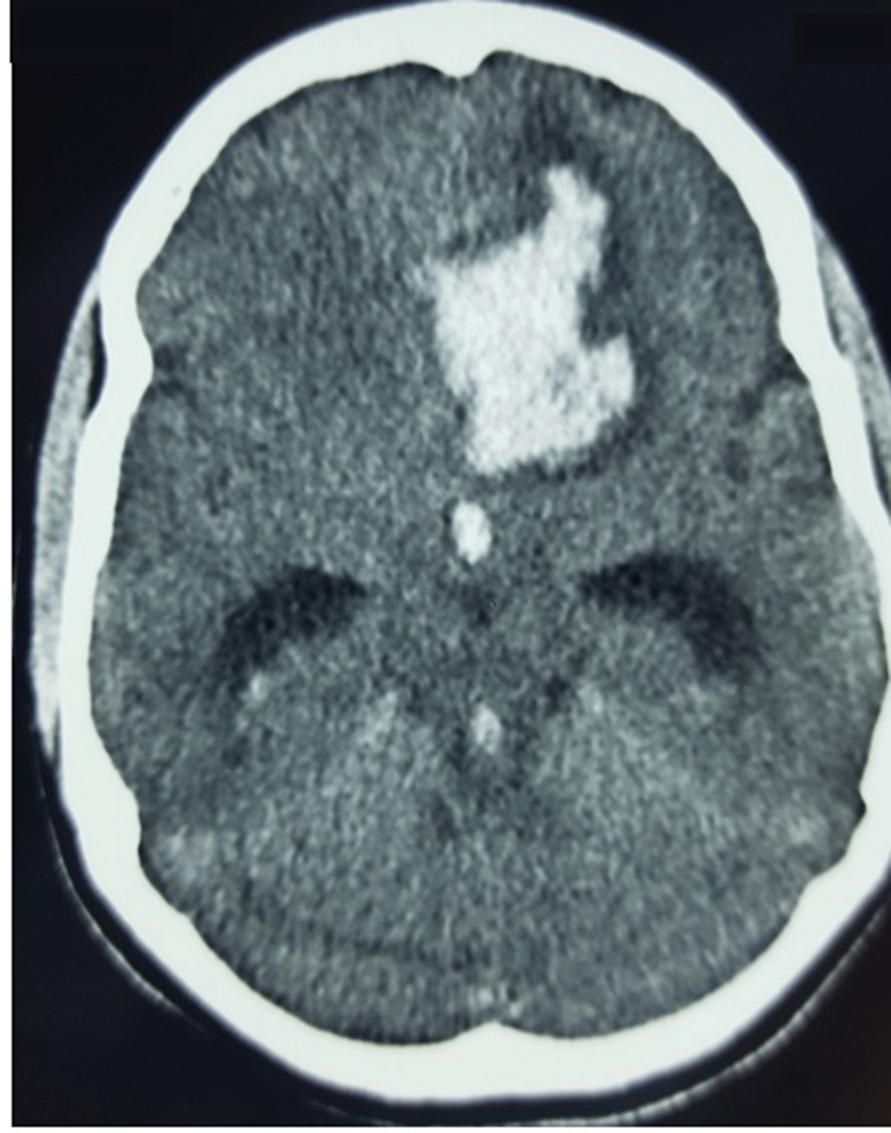
Axial CT scan after second bleed demonstrating left frontal haematoma opened to ventricular system with signs of acute hydrocephalus.

**Figure 4.**
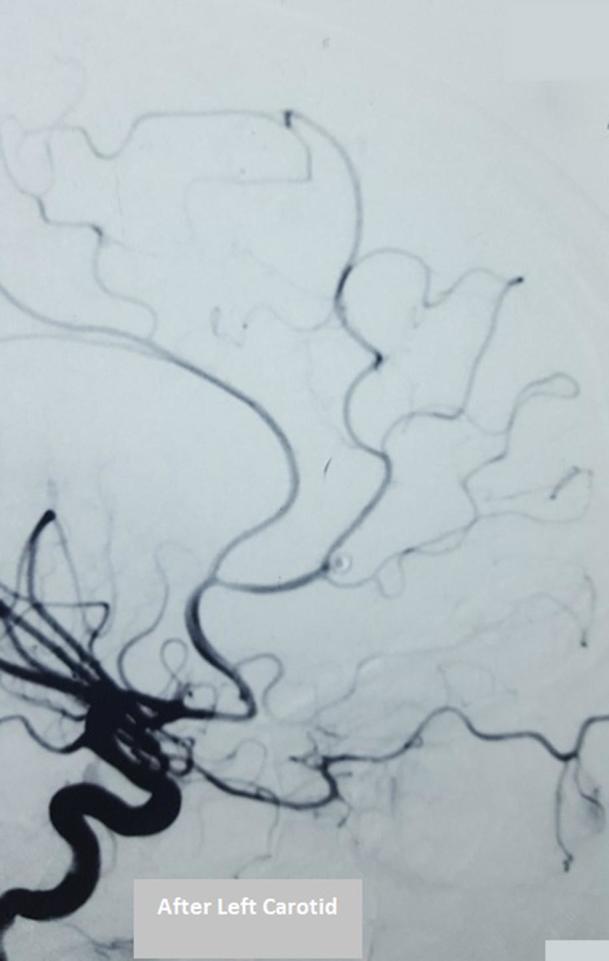
Post-embolization left carotid angiograms lateral view demonstrating complete aneurysm obliteration and distal arterial lumen totally permeable.

**Figure 5.**
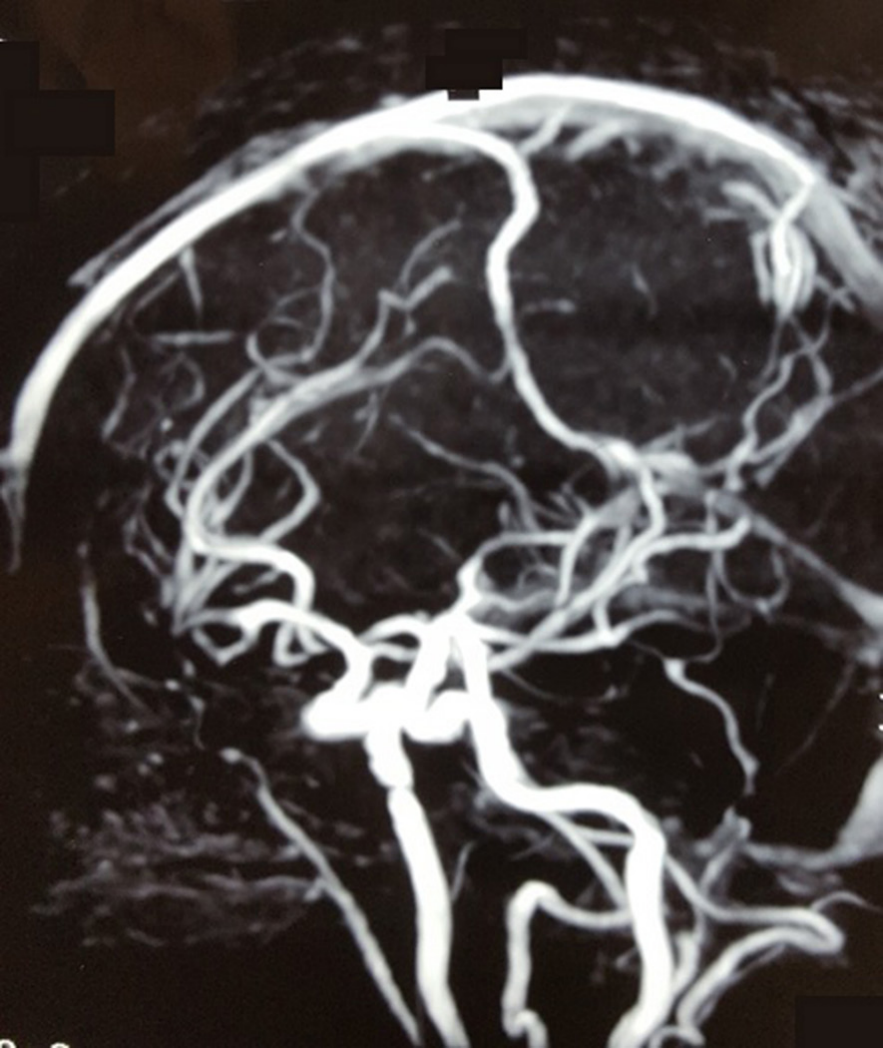
Follow-up MRA brain after 6 months showing absolute closure of FPA aneurysm. No recanalization is evident. FPA, frontopolar artery; MRA, magnetic resonant angiogram.

**Figure 6.**
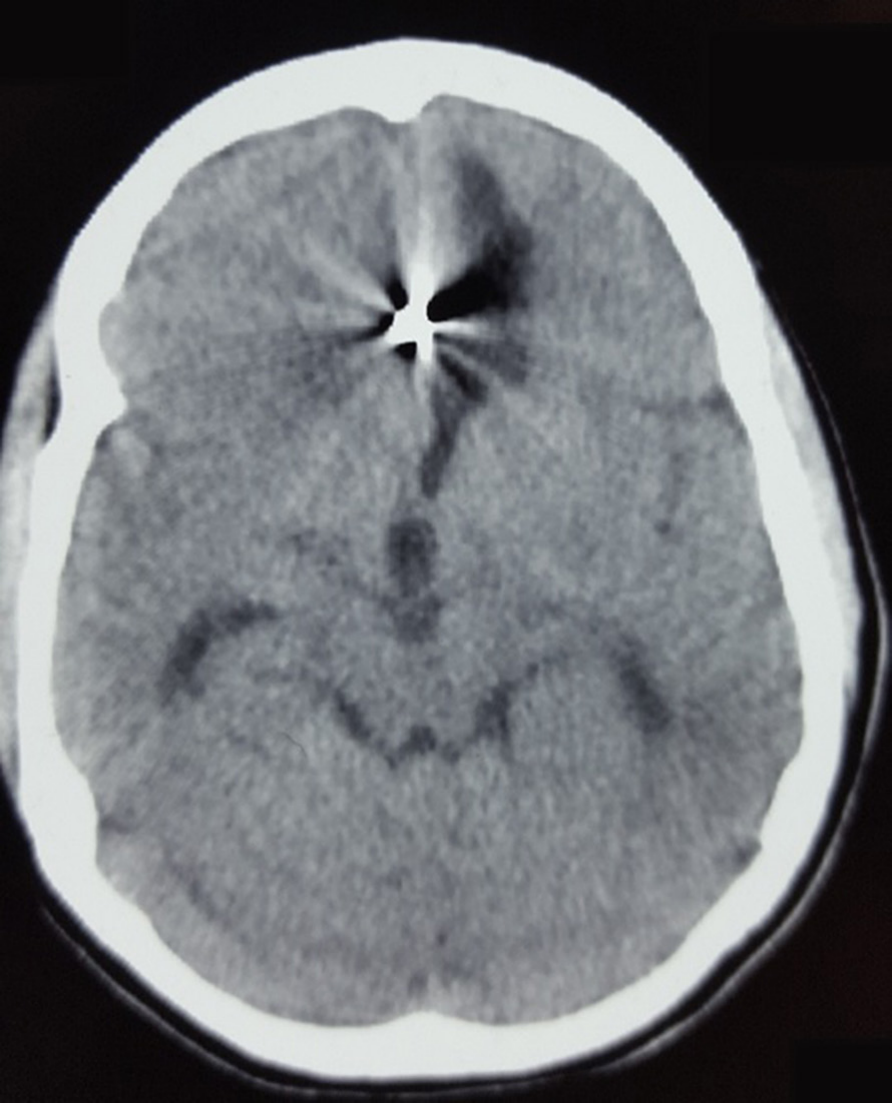
Axial CT scan showing metallic artefact due to coil along with chronic infarct frontal lobe.

A 17-year-old girl was admitted after experiencing sudden onset of thunderclap headache preceded by acute loss of consciousness at her home. There was no past history of hypertension, seizures, drug abuse, trauma or infection. On admission, the initial clinical examination showed an unconscious patient GCS 10/15 Hunt & Hess classification Grade IV. After stabilization, a CT scan was performed (Figure 1) showing diffuse subarachnoid haemorrhage and cisternal clot at interpeduncular cistern (Modified Fisher I). Moderate dilatation of temporal horns of lateral ventricle also seen. Angiography (Figure 2) revealed a ruptured saccular aneurysm of less than 3 mm in diameter, with a neck of 1.5 mm at the origin of left frontopolar artery (FPA). Considering the small size of aneurysm and ruptured nature, we decided to manage it conservatively. After 2 weeks of initial subarachnoid haemorrhage, the aneurysm bled once again. Another CT scan (Figure 3) was obtained showing superior interhemispheric frontal haematoma opened to ventricular system (Modified Fisher IV). Coiling done on Day 15 after second haemorrhage. After selective microcatheteration of the left FPA aneurysm, it was completely obliterated with a single coil maintaining the distal arterial lumen totally permeable (Figure 4). Follow-up magnetic resonant angiogram was performed 6 months after coil embolization and showed stable occlusion. (Figure 5). The patient never lost follow up, after 1 year of coiling she had an episode of GTCS, immediate CT scan done showing chronic infarct left frontal lobe (Figure 6).

## Discussion

According to researchmost ACA aneurysms occur at the mean age of 55 years. Only 1200 cases have been reported between 1939 and 2011. The majority of these are proximal internal carotid artery aneurysms and distal anterior cerebral artery’s are even rarer. The ultimate reason behind this is unknown; however, it has been postulated that distal anterior cerebral artery are associated with anatomical variations at this site. It is further posited that supreme anterior communicating artery is a communication between the two pericallosal arteries at the major branches during embryonic life. This connection may be a remnant and could be the aetiology of aneurysms at this site.^6^

Conflicting accounts of literature put the occurrence of DACA aneurysms between 2.1 and 9.2% of all intracranial aneurysms.^7, 8^ Of all the aneurysms in the DACA region, those in the A3 segment are most common with an incidence rate between 69 and 82%, mostly concentrated at the CMA-PCA (callosomarginal artery-pericallosal artery) junction.^9^ In the A2 segment, incidence reports are between 5 and 22% of all DACA aneurysms. These happen mainly at the A2 trunk or its frontonasal braches having the highest concentration at A2A on A2 truck at the origin of the FPA.^10^

DACA aneurysms due to their rarity, complex morphology, small size and depth inside the brain have always been a challenge for surgeons with high morbidity rates.

Open surgery, when using clip reconstruction or *in situ* bypass with aneurysm trapping receives high occlusion rates but the periprocedural morbidity and mortality rates are still quite high.^11, 12^

Endovascular coil embolization allows for a complete to near complete aneurysm occlusion in 80% of cases. Since the advent of modern research and development of newer more suitable microcatheters, guidewires and coils DACA aneurysms can be treated with far higher success rates.

It is very important to differentiate between saccular and dissecting aneurysms when choosing modality of treatment and as such we must first correctly identify the aneurysm. Dissecting aneurysms rarely develop near secondary to dissection of the intracranial arteries and are mostly found in the posterior circulation of the vertebrobasilar system with a close association to subarachnoid haemorrhage. Whereas, those of the carotid system are usually presented with cerebral infarctions caused by stenosis and occlusion. Only 14% of anterior circulation aneurysms are dissecting in nature and present as cerebral infarction and not as haemorrhage. Lastly, dissecting aneurysms tend to occur proximally in the anterior circulation because there is a stronger arterial pressure present there compared with the distal branches.

Saccular aneurysms are almost always the result of hereditary weakness in blood vessels and typically occur in arteries of *“circle of Willis”*. They lack a tunica media and elastic lamina around their dilated location with wall of sac made up of thickened hyalinized intima and adventitia. These are mostly found around the circle of Willis at bifurcations where small vessels link to main vessels because the brain vasculature is inherently weak at these points. Making them particularly susceptible to saccular aneurysms.

**Figure 7.**
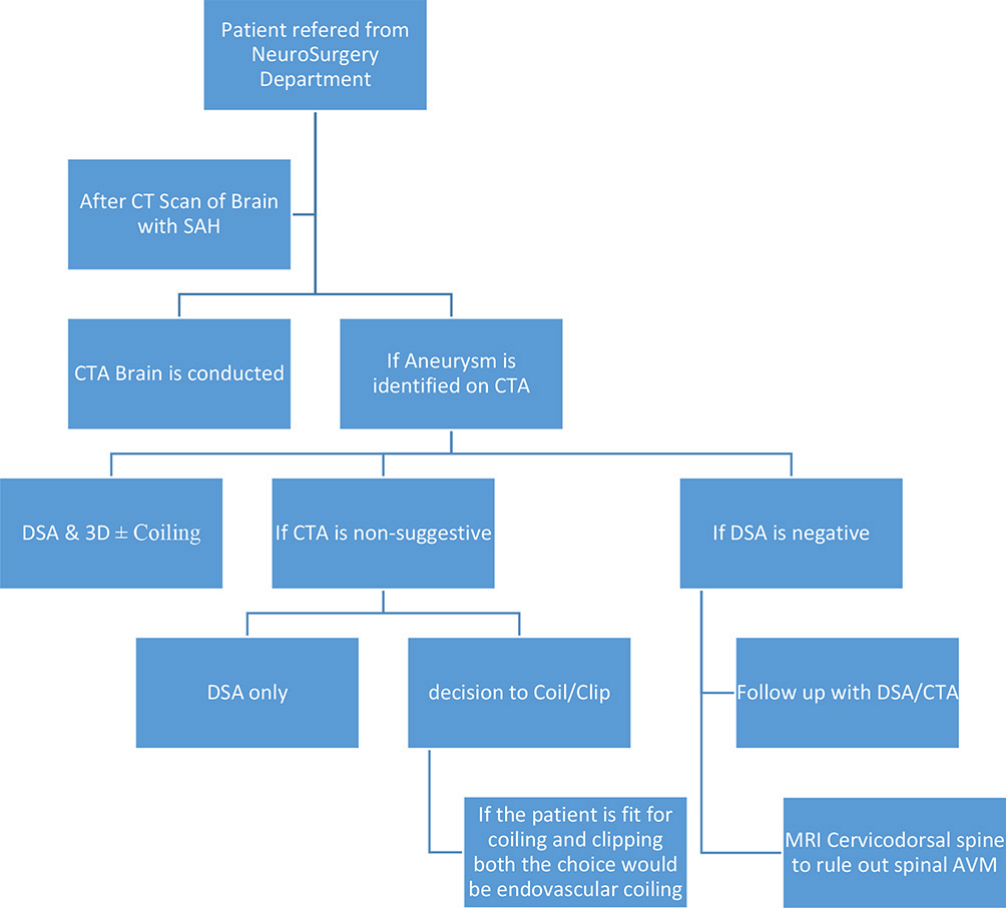
Workflow to decide treatment of ruptured intracranial aneurysm

When choosing treatment modality, the patient and aneurysm characteristics need to be taken into account to formulate the best treatment option varying on a case to case basis and giving preference to coiling rather than surgery. Table 1 offers a complete breakdown of the key deciding factors and a guide on how to choose preferred treatment modality. (Figure 7) outlines a flow-chart for how the treatment modality should best be carried out. In this case, the aneurysm was successfully obliterated using coiling and gives a prime example of successful treatment of DACA aneurysms using an endovascular approach.

**Table 1.** Factors to decide whether to clip or coil

**Factor**	**Details**	**Coiling ** **possible**	**Clipping ** **possible**	**Preferred**
Age	Young age	Yes	Yes	Clipping
Old age	Yes	No	Coiling
GCS	10 and below	Yes	No	Coiling
Above 10	Yes	Yes	Clipping
Co- morbidities	Present	Yes	No	Coiling
Absent	Yes	Yes	Clipping
Location	Anterior circulation	Yes	Yes	Clipping
Posterior circulation	Yes	No (possible with great difficulty and poor outcome)	Coiling
Size	Small	Yes	Yes	Coiling
Large	Coiling with assistant techniques	No	Coiling
Type	Dissecting	Yes	Yes	Coiling
Saccular	Yes	Yes	Coiling
Finances	Present	Yes	No	Coiling
Absent	No	Yes	Clipping

## Conclusion

Despite the anatomical difficulty of access and pathological hindrance of small aneurysms that result in relatively high intraprocedural events, the outcome still remains favourable in the present case. Endovascular technical evolution and the skill of an experienced neurointerventionalist make endovascular management of small DACA aneurysms a feasible alternative.

## Learning Points

1. DACA aneurysms are very rare with only 1200 cases reported since 1939. While there is debate on their true cause, it is posited that they are a result of embryonic remnants of supreme anterior communicating artery.
2. Dissecting aneurysms are mainly found near secondary to dissection of the intracranial arteries and in the posterior circulation of the vertebrobasilar system with stenosis and occlusion. Saccular aneurysms are mainly found around the circle of Willis at the bifurcation of small vessels because the brain vasculature is weakest at those points.
3. There is a huge risk in the surgical approach to such cases, as it delves too deep into the brain often resulting in a poor outcome, especially in third world countries; thus, an endovascular approach should be taken given that it offers better results^13^ . 
